# Characterization of Mutational Signatures in Tumors from a Large Chinese Population

**DOI:** 10.1158/2767-9764.CRC-24-0496

**Published:** 2025-08-29

**Authors:** Aaron Chevalier, Tao Guo, Natasha Q. Gurevich, Jingwen Xu, Masanao Yajima, Joshua D. Campbell

**Affiliations:** 1 Section of Computational Biomedicine, Department of Medicine, Boston Unversity Chobanian & Avedisian School of Medicine, Boston, Massachusetts.; 2 Bioinformatics Program, Boston University, Boston, Massachusetts.; 3 Department of Mathematics & Statistics, Boston University, Boston, Massachusetts.

## Abstract

The majority of mutational signatures have been characterized in tumors from American and European countries, and the degree to which mutational signatures are similar to or different from those in Chinese populations has not been fully explored. We leveraged a large-scale clinical sequencing cohort of tumors from the Chinese population developed by OrigiMed (OM). A total of 2,115 tumors from 25 major tumor types that had at least 10 single-base substitutions (SBS) were used for mutational signature analysis. Sixteen mutational signatures from the Catalogue of Somatic Mutations in Cancer (COSMIC) database were identified in the OM cohort using the musicatk package. The activity levels for most of the signatures were similar to those in a cohort from Memorial Sloan Kettering in the United States. The activity level for the apolipoprotein B mRNA editing catalytic polypeptide-like–related signature SBS2 was significantly lower in breast tumors in the OM cohort. The aristolochic acid signature SBS22 was observed in four soft-tissue sarcomas, and the *POLE*-associated signature SBS10 was observed in a gallbladder carcinoma. In lung adenocarcinoma, the polycyclic aromatic hydrocarbon signature SBS4 was significantly higher in males compared with females but not associated with smoking status. UV-related mutations were significantly lower in cutaneous melanomas from the Chinese cohort compared with the American cohort and may contribute to lower response rates to immunotherapy observed in this population. Overall, these results add to our understanding of the mutational processes that contribute to tumors from the Chinese population.

**Significance:**

Analysis of a large Chinese cohort from 25 tumor types reveals similarities and differences in the activity of mutational signatures compared with other populations.

## Introduction

A variety of exogenous exposures or endogenous biological processes can contribute to the overall mutational load observed in human tumors (1–4). Many different mutational patterns, or “mutational signatures,” have been identified across different tumor types (2, 5, 6), which can provide a record of environmental exposure or clues about the etiology of carcinogenesis (7). The majority of mutational signature characterization has been performed using tumors from American and European populations due to the availability of sequencing data in these regions from large-scale atlases, such as The Cancer Genome Atlas (8) and the International Cancer Genome Consortium (9). Although cancer incidence and mortality can vary across regions and countries (10), the degree to which mutational signatures are similar to or different between populations remains an open area of exploration. Exposures to certain carcinogens and their corresponding mutational signatures have been characterized in Eastern populations. For example, aristolochic acid is found in herbal medicines used in Asian populations and can cause the single-base substitution (SBS) signature SBS22 in hepatocellular carcinomas (HCC; ref. 11). Similarly, exposure to aflatoxin B1, produced by molds growing on food, can cause SBS24 in HCCs (12). Although some unique mutational signatures have been identified in various Eastern populations, mutational signatures and their activity in tumors from Chinese patients have not been well characterized across cancer types.

Previously, a large-scale cohort of tumors from the Chinese population was profiled with a targeted DNA sequencing panel containing 450 genes developed by OrigiMed (OM; ref. (13). This work compared the frequency of driver genes, tumor mutational burden (TMB), gene fusions, and clinically actionable alterations between tumors from Chinese and American populations. A total of 31 ethnicities were presented in the cohort, with Han being the most frequent (92%). The majority of patients in the study were from eastern and southern provinces in China (41% and 29%, respectively). The frequencies of nonsynonymous mutations in driver genes were largely similar across populations although some differences were observed in certain tumor types, such as lung adenocarcinoma (LUAD) and hepatobiliary tumors. Clinically actionable genomic alterations were found in 64% of cancers in the OM cohort and were consistent with those found within the American cohort. In contrast, significant differences in TMB were observed between the Chinese and American cohorts in nearly half of the cancer types examined. Despite the differences in overall mutational burden, mutational signatures were not examined or compared across populations.

We further leveraged the OM cohort to characterize the landscape of highly active mutational signatures across a large spectrum of tumor types in the Chinese population. Quantification of activities for 16 signatures revealed that the majority of signature activities were not significantly different between tumors from Chinese and American populations. However, different patterns of associations between patient characteristics and activities were found for exposure-related signatures such as SBS4 (smoking) and SBS7 (UV). Overall, these findings further elucidate the patterns of mutational signatures and their association with clinical features across diverse regions and populations.

## Materials and Methods

### Cohort

The full details of the patient consent, clinical characteristics, biospecimen processing, and DNA sequencing have been previously published (13). Briefly, tumors from 25 tumor types were profiled with a DNA sequencing panel targeting 450 genes, *TERT* promoter mutations, and 39 introns in a Clinical Laboratory Improvement Amendments–certified and College of American Pathologists–accredited laboratory by the China-based company OM. Each tumor has a major cancer type and a more specific detailed cancer type. For most tumors, we used the major cancer type label. Given the high numbers of non–small cell lung cancers (NSCLC) in this dataset, we divided this group into LUADs, lung squamous cell carcinomas (LUSC), and “NSCLC other” that contained the subcategories of “lung adenosquamous carcinoma,” “large cell lung carcinoma,” and “NSCLC other.”

### Signature discovery and prediction

SBSs were extracted in each tumor to produce a count table using the musicatk R package version 1.91 (14). Mutational signature discovery and prediction were limited to 2,115 tumors from 25 major tumor types that had at least 10 SBSs. Mutational signatures were first discovered *de novo* using nonnegative matrix factorization from the SigProfiler package version 1.1.4 (15), setting the reference genome to GRCh38. The number of signatures predicted varied from one to eight, and the optimal number of six was chosen based on the maximal difference between the mean sample cosine distance and average stability metrics. Discovered signatures were compared with those from the Catalogue of Somatic Mutations in Cancer (COSMIC) version 2 database using cosine similarity. Next, the activity of all COSMIC version 2 signatures was predicted using the “auto_predict_grid” function in the musicatk package, with the “algorithm” parameter set to “lda_posterior” and the “sample_annotation” parameter set to the tumor type (14). Initially, 25 signatures were detected with this method. We manually reviewed each signature according to criteria set by the pan-cancer analysis of whole genomes mutational signature working group (4). Specifically, we examined the mutation counts from individual tumors with high predicted activities of each signature. If no individual tumors displayed a mutational pattern that was predominantly correlated with the signature, then this signature was excluded from the final signature set. The final signature activities were predicted using the “predict_exposure” function with the “algorithm” parameter set to “lda” and specifying 16 SBS signatures that passed inspection, including 1, 2, 4, 5, 6, 7, 10, 13, 15, 17, 18, 20, 21, 22, 24, and 26. A signature was considered highly active in an individual tumor if it contained at least 10 estimated counts in that tumor. Proportional signature activities were calculated by dividing each signature activity within a tumor by the total number of estimated signature activity counts from that tumor.

### Comparison with an American cohort

Mutations for the Memorial Sloan Kettering (MSK) cohort were downloaded from cBioPortal (https://www.cbioportal.org/study/summary?id=msk_impact_2017; RRID:SCR_014555). SBSs were extracted from each tumor using the musicatk package, and samples with at least 10 mutations were retained for the analysis. The same signatures that were detected in the OM cohort were predicted in the MSK cohort using the “predict_exposure” function with the “algorithm” parameter set to “lda.” Tumor types were mapped between cohorts using either the major or more detailed cancer-type label. The proportional signature activities were calculated for each tumor by dividing each signature activity by the total number of mutations for that tumor. Proportional signature activities were compared across cohorts to account for differences in coverage between the different targeted sequencing panels used for each study. The medial level of proportional activity for each signature in a tumor type was compared across cohorts using a Wilcoxon rank-sum test followed by correction for multiple hypothesis testing using the FDR. The frequencies of tumors with detected signature activity were compared across cohorts using Fisher’s exact test followed by an FDR correction. Only tumor types with at least five tumors in both cohorts were included in the analysis. For both comparisons, only signatures with a median level of 0.01 across all samples from both cohorts were included in the analysis.

### Determining associations of signature activities with other clinical or genomic variables

The Wilcoxon rank-sum test was used to assess differences in normalized signature activities between two groups. The association between SBS4 activity and covariates was also assessed using multivariate linear regression within each lung tumor type. SBS4 activity was log-transformed after adding a pseudocount of 1 and treated as the dependent variable. Independent variables included age at diagnosis, sex, smoking status, stage according to the American Joint Committee on Cancer, tumor purity, *EGFR* mutation status, and sample type (primary or metastasis). Smoking status included categories for “smokers” and “nonsmokers.” Samples with “unknown” status for any variable were excluded from the regression, and any variable that did not have more than one category within a cancer type was excluded from the regression for that cancer type. To determine associations with chemotherapy status, we used a linear model that had the log-transformed signature activities as the dependent variable and age and chemotherapy as independent predictor variables. This analysis was performed in tumor types with at least five patients who received chemotherapy. *P* values for the chemotherapy term were corrected for multiple hypothesis testing using the FDR.

### Data availability

The mutations and clinical data analyzed in this study were retrieved from the cBioPortal (https://www.cbioportal.org/study/summary?id=pan_origimed_2020; RRID:SCR_014555). All codes for the analysis are available on GitHub at https://github.com/campbio-manuscripts/Chinese_Mutsigs. Any additional data generated in this study are available from the corresponding author upon request.

## Results

### Characterization of highly active mutational signatures in tumor from Chinese patients

Mutational profiles for 10,194 tumors were obtained from the cBioPortal with relevant clinical data. The clinical characteristics of the full cohort have been previously described (13) and are summarized in Supplementary Table S1. A total of 2,115 tumors with at least 10 mutations from 25 tumor types were utilized for mutational signature analysis. Fifty-five percent of patients had advanced-stage cancers (stage III/IV), whereas 34% had early-stage cancers (precancers or stage I/II). The majority of patients were treatment-naïve (74%). We first performed *de novo* deconvolution with nonnegative matrix factorization using the SigProfiler package (15) and identified six mutational signatures (Supplementary Fig. S1; Supplementary Tables S2 and S3). All signatures were highly correlated with previously defined signatures in the COSMIC database, indicating that no new highly active mutational processes were present in this cohort. The discovered signatures included those correlated with SBS1/6, SBS4, SBS10, SBS12/26, SBS2/13, and SBS22.

**Figure 1:**
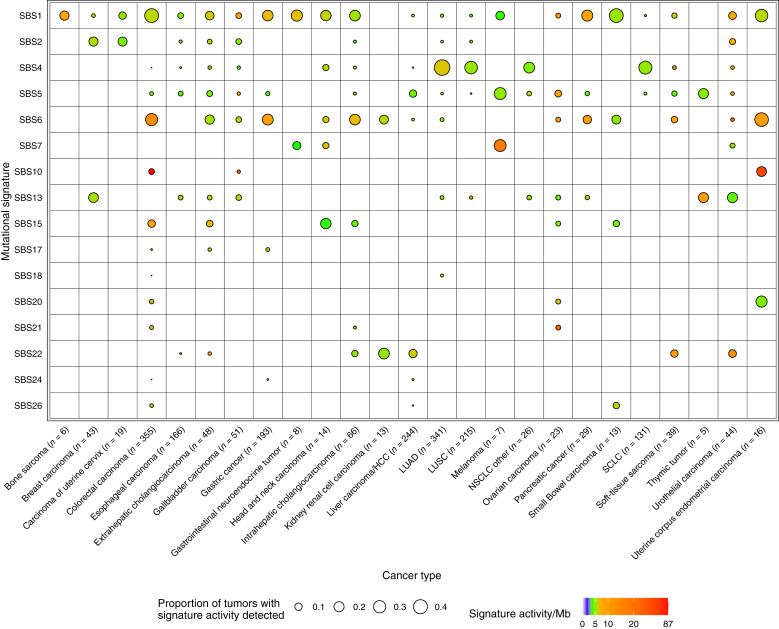
The landscape of highly active mutational signatures in a large Chinese population. Sixteen mutational signatures were identified across 2,115 tumors from 25 tumor types. The size of the dot corresponds to the percentage of tumors within each tumor type that have detectable levels of the signature. Signatures with at least 10 counts were considered detected in an individual tumor. The color of the dot corresponds to the median activity of each signature within the detected samples per megabase (Mb).

The limited number of mutations in the targeted sequencing data can hinder *de novo* mutational signature discovery. Therefore, we also predicted signature activity levels for existing COSMIC signatures using the musicatk package (Supplementary Table S4; ref. 14). The landscape of activities for 16 signatures across tumor types is shown in Fig. 1 and listed in Supplementary Table S5. Signatures related to endogenous biological processes included the aging-related signature SBS1 and the clock-like signature SBS5, which were broadly detected across tumor types. The apolipoprotein B mRNA editing catalytic polypeptide-like–related signatures SBS2 and SBS13 were often observed together across a variety of epithelial tumor types, such as breast carcinoma, carcinoma of the uterine cervix, esophageal carcinoma, gallbladder carcinoma, NSCLC, and urothelial carcinoma. Several signatures related to defective mismatch repair, microsatellite instability, or defective DNA polymerase activity were detected, including SBS6, SBS15, SBS20, SBS21, SBS26, SBS17, and SBS10. As previously observed in American and European cohorts, these signatures often co-occurred in the same samples (1, 4). One or more of these signatures were active in samples from many of the same tumor types as other cohorts, including bone sarcoma (1), colorectal carcinoma (1), intra- and extrahepatic cholangiocarcinoma (1), gallbladder carcinoma (16), gastric cancer (17), head and neck carcinoma (1), kidney/renal cell carcinoma (1), ovarian carcinoma (1), pancreatic cancer (1), small bowel carcinoma (18), and uterine corpus endometrial carcinoma (1). We also observed three gallbladder carcinomas with high levels of SBS6, as well as one sample with SBS10 (*POLE*), which has not been previously observed for this cancer type (19, 20). High levels of SBS17 were found in a small number of tumors from breast carcinoma, colorectal carcinoma, extrahepatic cholangiocarcinoma, gastric cancer, or liver carcinoma/HCC. Although this signature often co-occurred with other signatures, some tumors contained only this signature. High levels of SBS10 related to defects in *POLE* were observed in 22 colorectal carcinomas and three uterine corpus endometrial carcinomas.

**Figure 2:**
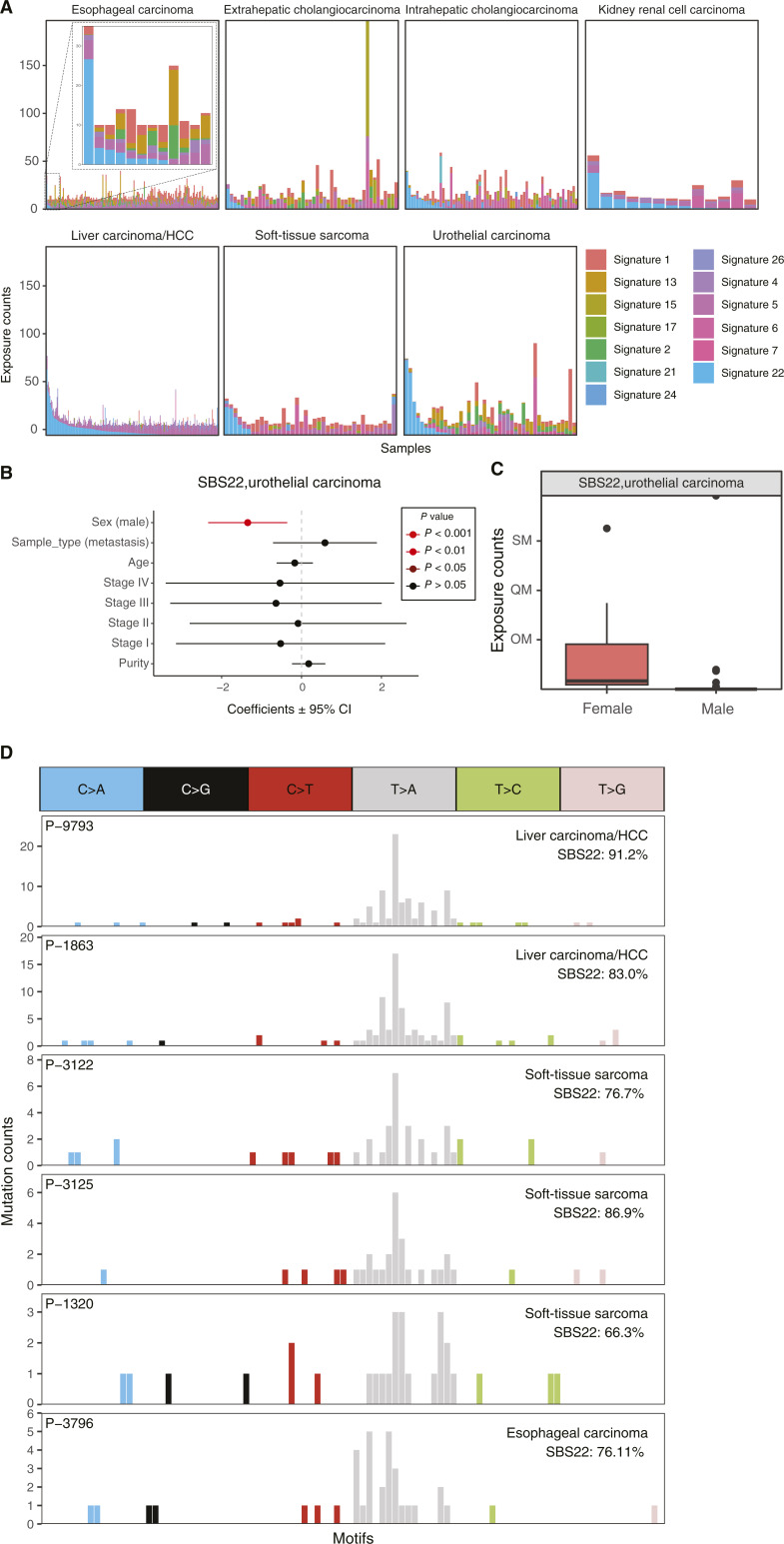
Prevalence of aristolochic acid (SBS22) activity across tumor types. **A,** SBS22 reflects exposure to aristolochic acid and was highly active in 32 liver carcinomas/HCCs, six intra- or extrahepatic cholangiocarcinomas, three kidney renal cell carcinomas, and five urothelial carcinomas. This signature was also detected in four soft-tissue sarcomas and one esophageal carcinoma, which has not been reported in American and European cohorts. Each bar represents the level of the estimated signature activities in each sample, and samples are ordered by the level of SBS22. **B,** Using a multivariate linear model, SBS22 activity was associated with sex in urothelial carcinoma. **C,** SBS22 activity levels in urothelial carcinoma were higher in females compared with males. **D,** Mutation counts for two example liver carcinomas/HCCs, three soft-tissue sarcomas, and one esophageal carcinoma that predominantly contain the SBS22 signature. CI, confidence interval.

Several detected signatures are known to be caused by exposure to exogenous DNA-damaging agents. SBS4 is caused by exposure to polycyclic aromatic hydrocarbons (PAH) such as benzo[a]pyrene in cigarette smoke (24) and was observed in small cell lung cancers (SCLC) and NSCLCs. SBS22 is caused by exposure to aristolochic acid and was detected in liver carcinoma/HCCs, intra- and extrahepatic cholangiocarcinomas, kidney/renal cell carcinomas, and urothelial carcinomas (Fig. 2A). Interestingly, SBS22 was also detected in four soft-tissue sarcomas and one esophageal carcinoma, which has not been previously reported (Fig. 2D; refs. 21, 22). SBS22 activity in urothelial carcinoma was higher in females than in males (*P* < 0.001; Fig. 2B and C). No significant associations with clinical variables were found in other tumor types. SBS24, reflecting aflatoxin B1 exposure, was detected at high or moderate levels in two liver carcinoma/HCCs, similar to previous reports (1, 23). This signature was also highly detected in one colorectal carcinoma and one gastric cancer, raising the possibility that this exposure, or something similar, can induce mutations in more tumor types than previously appreciated. SBS7 is caused by UV radiation exposure and was highly detected in two melanomas, one head and neck carcinoma, and two urothelial carcinomas. SBS18, which is potentially due to oxidative stress, was detected in one colorectal carcinoma. Although some patients received chemotherapy, no chemotherapy-associated signatures from COSMIC were detected. We also did not observe significant differences in the activity levels for any signature between samples with and without chemotherapy in any tumor type (FDR >0.05).

### Comparison of signature activities between Chinese and American cohorts

**Figure 3:**
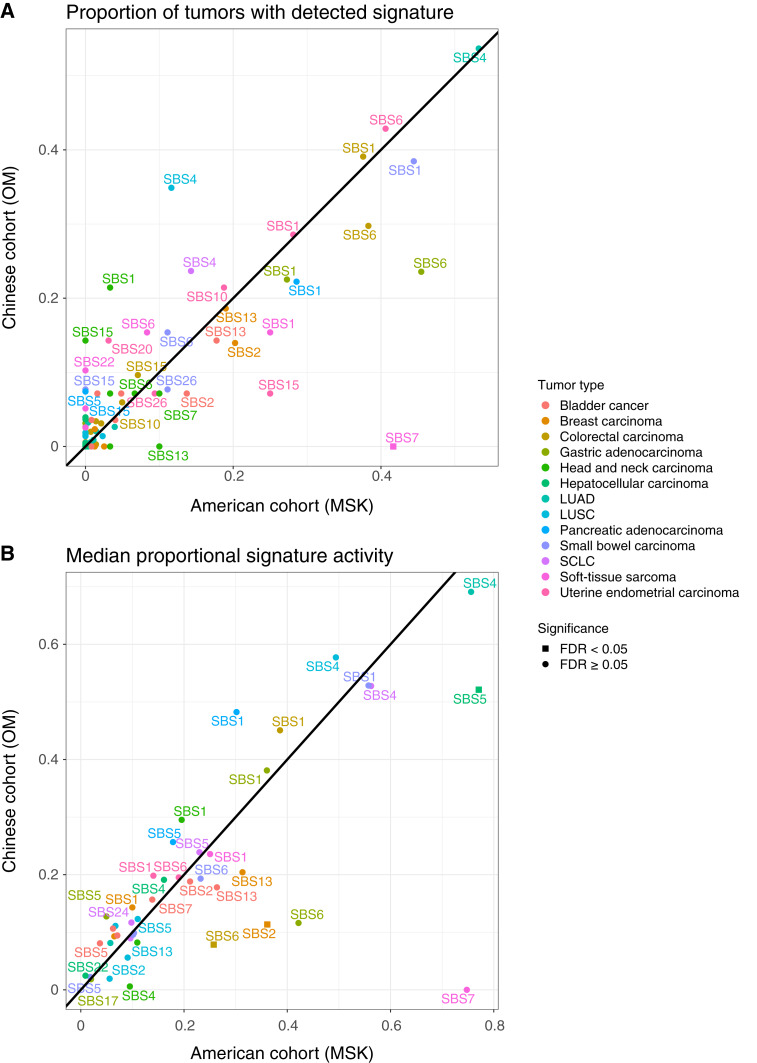
Comparison of signature detection and activity levels across populations. **A,** Signatures with at least 10 counts in a tumor were considered detected in that tumor. The frequencies of detected signatures were compared across Chinese (OM) and American (MSK) cohorts using Fisher’s exact test with an FDR correction. Only tumor types with at least five tumors in each cohort were included. The majority of signature detection rates were not significantly different between cohorts, with the exception of the UV-associated signature SBS7, which was found in a higher proportion of soft-tissue sarcomas in the MSK cohort compared with the OM cohort. **B,** The median levels of proportional signature activities were compared for matched tumor types between the Chinese (OM) and American (MSK) cohorts using a Wilcoxon rank-sum test with an FDR correction. The majority of signature activities were not significantly different between cohorts with the exception of the apolipoprotein B mRNA editing catalytic polypeptide-like SBS2 signature in breast carcinoma, the SBS6 signature in colorectal carcinoma, and the SBS5 signature in HCC.

We next sought to directly compare the signature activity levels in this Chinese population to a similar clinical cohort of tumors from an American population. Specifically, we leveraged a dataset generated at MSK that was profiled with the Integrated Mutation Profiling of Actionable Cancer Targets targeted sequencing panel. After mapping major tumor types and tumor subtypes between cohorts, 13 groups with at least five samples in each cohort were compared (Supplementary Table S6). For each signature, we first compared the proportion of samples with high activity of that signature between cohorts using a Fisher’s exact test. The only significant difference was that the American cohort had a higher proportion of tumors with the UV signature SBS7 in soft-tissue sarcoma compared with the Chinese cohort (FDR <0.05; Fig. 3A; Supplementary Table S7). The MSK cohort did not have any tumors with high SBS22 reflecting that aristolochic acid exposure largely occurs in Asian populations. Next, we compared the median activity of the proposed signature activities across cohorts. The majority of the proportional signature activities were not significantly different between cohorts after applying an FDR correction (Fig. 3B; Supplementary Table S8). The only exceptions were that SBS2 in breast carcinoma, SBS6 in colorectal carcinoma, and SBS5 in HCC had significantly lower levels in the OM cohort compared with the MSK cohort (Wilcoxon rank-sum test; FDR <0.05).

### SBS4 activity is associated with sex in LUAD

**Figure 4:**
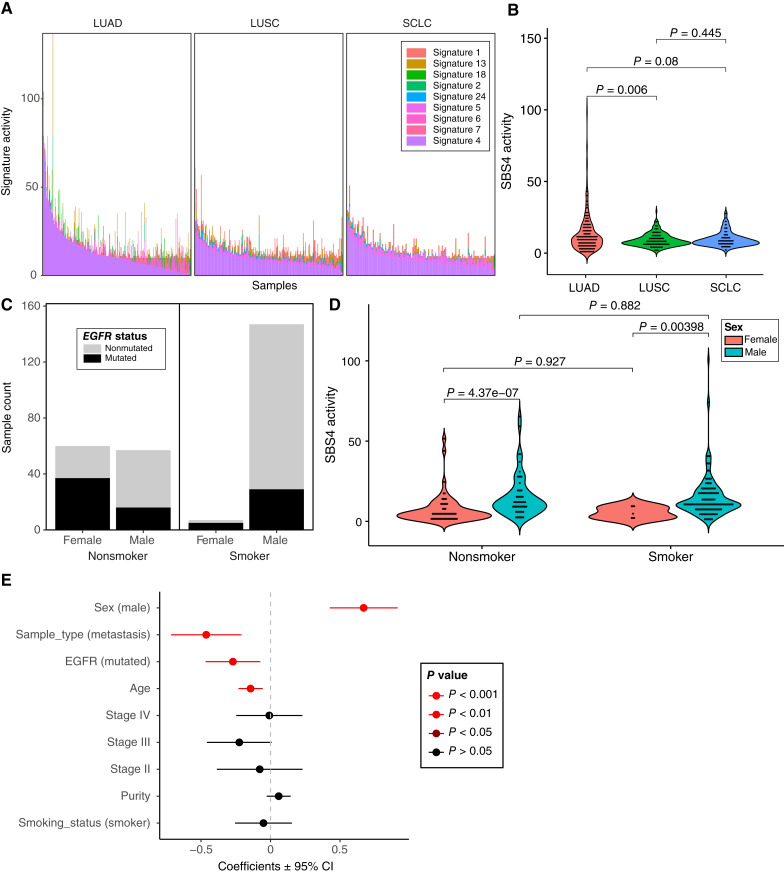
SBS4 activity associated with sex but not smoking status in LUAD. **A,** SBS4 activity was predominantly observed in lung cancers, including LUAD, LUSC, and SCLC. Each stacked bar represents the estimated activity levels of each signature in each tumor. Signatures with no estimated activity in these tumor types were excluded. **B,** SBS4 activity was compared between three major types of lung cancer using the Wilcoxon rank-sum test. LUAD had significantly lower SBS4 activity than LUSC. **C,** The relationship between smoking status, sex, and *EGFR* mutation status is shown for a subset of LUADs with complete clinical information (*n* = 271). A higher proportion of males were observed in smokers compared with nonsmokers, and a higher proportion of *EGFR* mutations were observed in females compared with males in both smokers and nonsmokers. **D,** Using a Wilcoxon rank-sum test, SBS4 activity was significantly higher in males compared with females in both smokers and nonsmokers but was not significantly different between smokers and nonsmokers within males or within females. **E,** Using a multivariate linear model, SBS4 activity was associated with sex, sample type (i.e., lower in metastasis compared with primary), *EGFR* mutation status, and age. CI, confidence interval.

SBS4 was highly prevalent across NSCLCs, including LUADs and LUSCs, as well as in SCLCs, as previously observed (Figs. 1 and 4A; refs. 1, 24–26). SBS4 activity was significantly lower in LUAD compared with LUSC (*P* = 0.006) but not significantly different between LUAD and SCLC or SCLC and LUSC (*P* > 0.05; Fig. 4B). In China, rates of cigarette smoking are much higher among males compared with females (27). We observed similar trends in this cohort, with a higher proportion of males among the smokers compared with the nonsmokers (*P* < 0.001; Fisher’s exact test; Fig. 4C). Furthermore, LUADs from female nonsmokers in Asian populations tend to be driven by alterations in *EGFR* (28). Similarly, in this cohort, we observed a higher proportion of tumors with *EGFR* mutations in females compared with males in both smokers (*P* = 0.0061) and nonsmokers (*P* = 0.0004; Fig. 4D). In American and European cohorts, SBS4 activity is strongly associated with smoking status in LUAD (29). Surprisingly, in this Chinese cohort, the SBS4 signature was not associated with smoking status but instead was strongly associated with sex (Fig. 4E). Specifically, the level of SBS4 activity was significantly higher in males compared with females among smokers (*P* = 0.00398) and even more so among nonsmokers (*P* = 4.37e−07). SBS4 was not significantly different between smokers and nonsmokers in males or between smokers and nonsmokers in females (*P* > 0.05), indicating that the association with sex is not just due to the differences in the prevalence of smoking between men and women. When applying a multivariable linear model to a subset of LUADs with complete clinical information (*n* = 271), SBS4 activity was significantly higher in males compared with females (*P* = 1.48e−07), decreased with age (*P* = 0.0013), was lower in tumors with *EGFR* mutations (*P* = 0.0072), and was lower in metastatic versus primary samples (*P* = 0.0004). SBS4 activity was not significantly associated with tumor purity, stage, or smoking status (*P* > 0.01; Fig. 4E). No strong associations were found between SBS4 activity and these variables in LUSC or SCLC (*P* > 0.01; Supplementary Fig. S2), demonstrating that this phenomenon is specific to LUAD. Sex-specific findings were still consistent after limiting the analysis to “oncogene-negative” LUADs (i.e., cases without mutations in the Ras/Raf/receptor tyrosine kinase pathway; refs. 29–31). Although this subset did not have female smokers (Supplementary Fig. S3A), the level of SBS4 activity was significantly higher in males compared with females among the nonsmokers (*P* = 0.00212), and SBS4 was not significantly different between smokers and nonsmokers in males (*P* = 0.301; Supplementary Fig. S3B). We also applied the same linear modeling procedure to the other signatures detected in LUAD. The only other moderate association between signature activities and clinical variables in LUAD was a lower level of SBS1 in males (*P* = 0.0256).

### Decreased SBS7 activity in cutaneous melanomas

**Figure 5:**
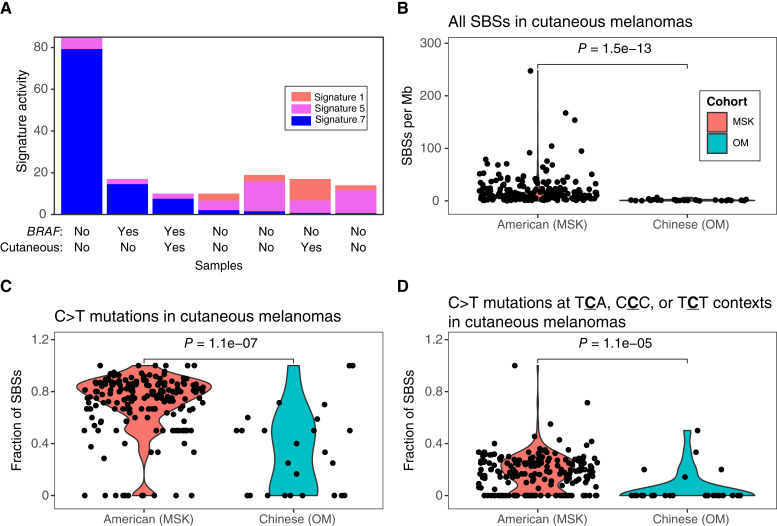
Lower activity of UV-associated mutations in cutaneous melanomas from Chinese patients. **A,** Bar plot of signature activities for seven melanomas in the OM cohort with at least 10 mutations that were included in the mutational signature analysis. **B,** The total number of SBS mutations per megabase (Mb), (**C**) the fraction of C>T mutations, and (**D**) the fraction of C>T mutations at the TCA, CCC, or TCT trinucleotide contexts were significantly lower in cutaneous melanomas from the Chinese cohort compared with cutaneous melanomas from the American cohort.

In the OM cohort, only 7 of 54 melanomas had at least 10 mutations (13%) and were included in the mutational signature analysis. This is significantly less than the MSK cohort, in which 145 of 358 (41%) of the melanomas had at least 10 mutations (*P* = 6.0e−05; Fisher’s exact test). Only two of those seven melanomas from the OM cohort had at least 10 mutations estimated to be from signature SBS7 (28.6%; Fig. 5A). In contrast, 118 of 145 tumors from the MSK cohort had at least 10 estimated SBS7 mutations (81%, *P* = 0.005; Fisher’s exact test). Asian populations have higher rates of acral and mucosal melanomas, whereas American and European populations have higher rates of cutaneous melanoma (32, 33). Acral and mucosal subtypes often have lower mutation rates and are less driven by UV-induced DNA damage compared with cutaneous melanomas (34). To understand differences between populations specifically in cutaneous melanoma, we expanded the analysis to examine the mutational profiles of all cutaneous melanomas across both cohorts (including tumors with less than 10 mutations, which were excluded in the mutational signature analysis). Cutaneous melanomas from the OM cohort (*n* = 26) had significantly lower SBS mutations per megabase than cutaneous melanomas from the MSK cohort (*n* = 191; *P* = 1.5e−13; Fig. 5B). They also had lower frequencies of any C>T mutations (*P* = 1.1e−7; Fig. 5C) and C>T mutations at the TCA, CCC, and TCT trinucleotide contexts, which are the most common contexts in signature SBS7 (*P* = 1.1e−5; Fig. 5D). The total SBS mutation rate or percentage of C>T mutations was not significantly associated with *BRAF* mutation status within the OM cohort (Supplementary Fig. S4). Overall, these results show that UV-associated mutation types are significantly lower in cutaneous melanomas from Chinese patients.

## Discussion

Overall, this analysis provides an overview of the mutational signatures that are highly active in a large Chinese population. When comparing this cohort with a similar American cohort, we found that the majority of signature activities were not significantly different between the populations. These results suggest that the levels of the common signatures are largely the same in tumors across Chinese and American populations despite the underlying heterogeneity in clinical and regional characteristics. SBS22 and SBS24 are known to be caused by exposure to carcinogens found in Asian regions (11, 12). Although these signatures were mainly detected in hepatobiliary tumors from the OM cohort, their activity levels were not statistically significantly different compared with the American cohorts. This is due to the overall relatively low numbers of tumors with detectable levels of these signatures within each tumor type (e.g., 3.9% for SBS22 and 0.3% for SBS24 in HCC). We did observe a significantly lower median proportion of the apolipoprotein B mRNA editing catalytic polypeptide-like–related signature SBS2 in breast cancers, SBS6 in colorectal carcinoma, and SBS5 in HCC in the OM cohort compared with MSK. The lower SBS2 activity in breast cancers contrasts with previous work analyzing data from tumors of patients from Hong Kong, which did not find significant differences in SBS2 or SBS13 between breast cancers from The Cancer Genome Atlas (35). The differences in signature activities between cohorts may be due to underlying differences in ancestral background. Frequencies of somatic alterations in some driver genes can vary across ancestral populations in cancers such as prostate and lung cancer (36, 37). This raises the possibility that rates of mutational signatures can also vary across ancestries although the mechanism of action for this is not understood. Another possibility is that rates of signatures could be affected by environmental factors, such as exposure to pollution or diet (38). Although this is known for certain signatures such as SBS4 (smoking) and SBS7 (UV), no associations with environmental variables have been described for SBS2, SBS5, and SBS6. Additional collection of environmental, dietary, and clinical risk factors on patients with sequencing data will be needed to elucidate the cause of these associations.

Similar to previous reports, SBS22 activity was identified in tumors from renal, liver, esophageal, and urothelial cancers (15). SBS22 is caused by aristolochic acid, a potent human carcinogen produced by *Aristolochia* plants used in herbal medicine, and has been linked to cancer and kidney disease (39). We report for the first time that this signature could also be identified in some soft-tissue carcinomas, suggesting that aristolochic acid exposure and herbal medicine usage could also contribute to this cancer subtype. We also observed an increase in the levels of SBS22 in females with urothelial carcinoma. This increase reflects the observation that females are associated with a higher risk of developing aristolochic acid–related urothelial carcinoma (40), which could potentially be explained by increased usage of herbal medicine among females in the Chinese population.

The SBS4 signature is enriched for C>A transversions and can be caused by the activation of PAH compounds. Similar to American and European cohorts, SBS4 signature activity was prevalent in the OM cohort in tumor types associated with smoke exposure, such as LUAD and LUSC. In contrast to other populations, SBS4 activity was not associated with smoking status in LUAD but was associated with sex, with males having significantly higher SBS4 activity than females in both smokers and nonsmokers. Inaccurate smoking status determined by self-report is one possible contributing factor to this discrepancy. However, if the smoking status is largely correct, then these results suggest that factors other than smoking status are contributing to SBS4 mutations in males from China. Smoking rates are considerably higher for men in China compared with women (52.1% vs. 2.7%; ref. 41). Other factors affecting SBS4 activity in males may potentially include a higher daily usage of cigarettes, a greater level of secondhand smoke, or a higher exposure to air pollution and ambient particulate matter compared with women (42, 43). Additionally, the impact of occupational exposure on lung cancer prevalence is expected to increase in China over the next several years (44). Other exposures associated with lung cancer include arsenic, asbestos, beryllium, cadmium, chromium, diesel engine exhaust, nickel, PAHs, and silica (45). Not all these carcinogens will produce a distinct mutational signature. Thus, other molecular approaches may be needed to identify exposure activity in tumor tissue. Additional cohorts of LUADs with highly detailed smoking and occupational exposure history will be needed to further understand the nature of this association.

We observed lower frequencies of UV-associated mutations in cutaneous melanomas in the Chinese cohort compared with the American cohort. In general, the prevalence of melanoma is 54 times lower in men and 60 times lower in women in China compared with the USA (46). Our findings corroborate and expand recent work showing that the number of UV-associated mutations in normal skin is lower in Asian populations compared with other populations despite Asian populations having higher levels of exposure to UV radiation (47). Lower response rates to immune checkpoint inhibitors have been observed in melanomas from the Chinese population compared with other populations (48), which could be a result of the lower TMB (49). Our data suggest that the lower prevalence of UV-associated mutations is a major contributor to the lower overall TMB in Chinese cutaneous melanomas.

One limitation of this study is that the OM cohort was profiled with a targeted sequencing panel, which has a lower number of mutations detected per tumor. Having lower counts can hinder the detection of signatures that tend to have lower activity levels. For example, we were not able to confidently detect signature SBS3, which denotes homologous repair deficiency caused by the loss of *BRCA1/2*. SBS3 has been previously characterized in breast cancers from Chinese and Korean populations (50, 51). Despite this limitation, we were able to detect signatures that are highly active in these tumors (i.e., signatures that produce enough mutations to be detected with a limited targeted sequencing panel) and characterize novel associations specific to this Chinese population. Although 294 samples in the OM cohort were from patients who received chemotherapy, known chemotherapy-associated signatures were not detected, suggesting that this was not a confounding factor in the dataset. Furthermore, we compared activity levels of each signature between samples that had received chemotherapy and those that did not. After FDR correction, no signature in any tumor type showed a significant difference between the activity levels of the chemotherapy and nonchemotherapy groups. Many patients may have received chemotherapy after the tumor tissue sample was obtained, and thus, the mutational profiles did not reflect exposure to chemotherapy agents. Additionally, although the patient population at MSK is predominantly White, some of the participants may have been of Asian descent. This may have decreased our statistical power to detect differences between cohorts. Lastly, the majority of analyses we performed were associative and exploratory in nature. Comprehensive profiling of tumors from Chinese patients using whole-exome or whole-genome sequencing will be needed to capture additional signatures and validate the associations we identified in this population.

### Conclusion

Mutational signatures have not previously been characterized in Chinese populations across a large number of tumors and tumor types. The results from our analysis demonstrate that many of the signatures and associations identified in American and European cohorts are also present in this Chinese population. Similarly, the major differences in the detected signatures are mostly related to exogenous exposures such as aristolochic acid and aflatoxin B1. Our analysis also revealed novel findings such as the presence of SBS22 in soft-tissue sarcomas and SBS10 in gallbladder carcinoma. Understanding the differences in SBS4 activity in LUAD related to sex and smoking status will need further exploration of the relationship between epidemiologic exposures and mutational patterns in this region. Additionally, the lower rates of SBS7 mutations in cutaneous melanomas may contribute to the lower response rates to immunotherapy observed in this population.

## Supplementary Material

Supplementary Tables Supplementary Tables

Supplementary Data Supplementary Data
