# Supplementary material for: Characterization of Mutational Signatures in Tumors from a Large Chinese Population
Source: Cancer Res Commun. 2025 Aug 29;5(8):1466–76. doi: 10.1158/2767-9764.CRC-24-0496 (PMC12395221; doi:10.1158/2767-9764.CRC-24-0496)
Supplement: Supplementary Data [file crc-24-0496_supplementary_data_suppsd.docx]

**Supplementary Figures**


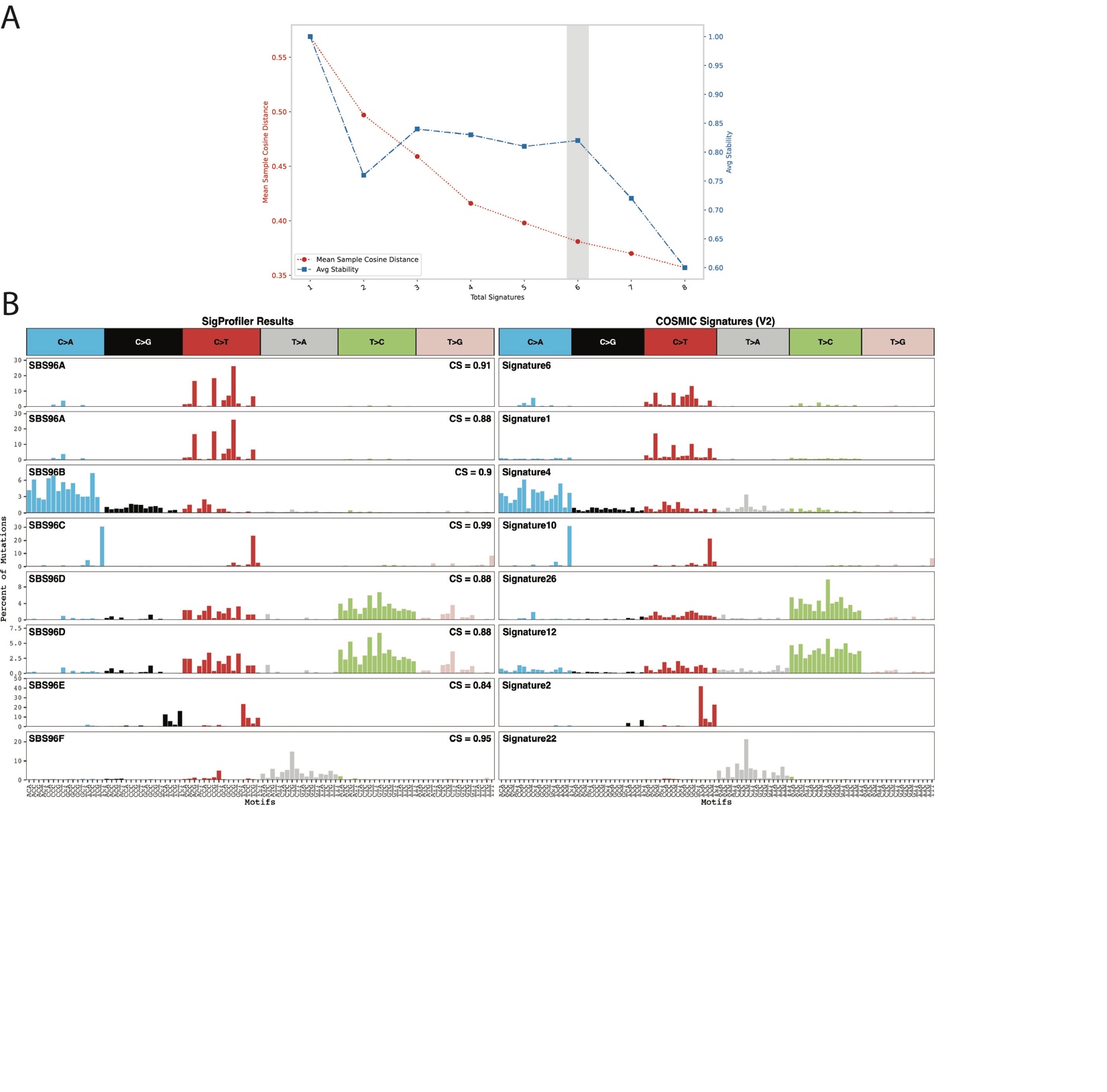


**Supplementary Figure 1. Discovery of mutational signatures *de novo* using NMF. (A)** NMF in the SigProfiler package was run to identify mutational signatures. The optimal number of signatures was determined to be six based on the maximal difference between the mean sample cosine distance and average stability metrics. **(B)** All discovered signatures were highly correlated with at least one known signature in the COSMIC database showing that no new highly active signatures could be identified in this cohort.

**
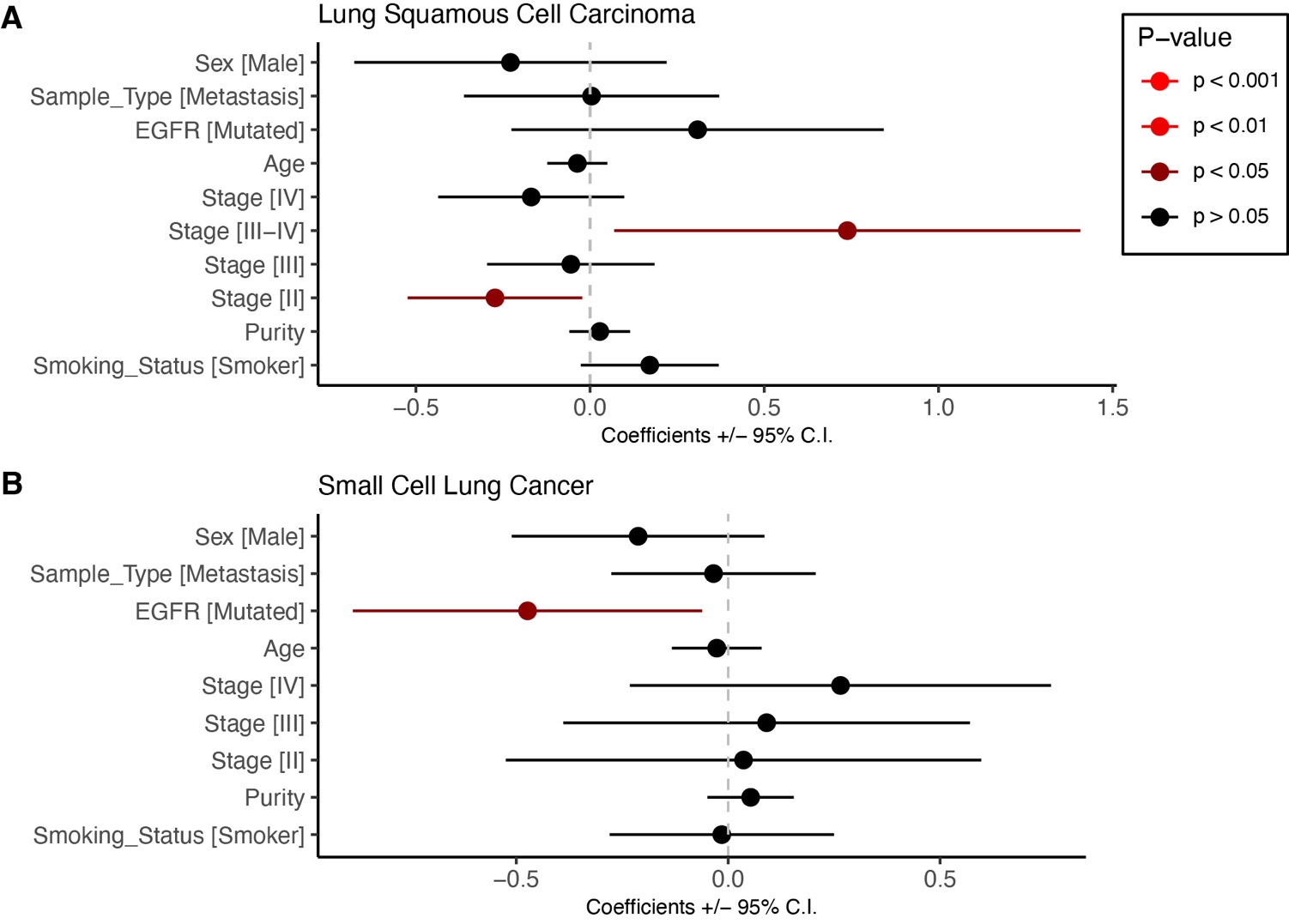
**

**Supplementary Figure 2. Lack of associations between clinical variables and SBS4 activity in lung squamous cell carcinoma (LUSC) and small cell lung cancer (SCLC).** A multivariate linear model was used to assess the relationship between SBS4 activity and clinical variables in **(A)** lung squamous cell carcinoma and **(B)** small cell lung cancer**.** Only moderate associations were observed between SBS4 activity and Stage II or Stage III-IV tumors in LUSC or *EGFR* mutations in SCLC (p < 0.05). No associations were observed between smoking status and sex (p > 0.05).

**
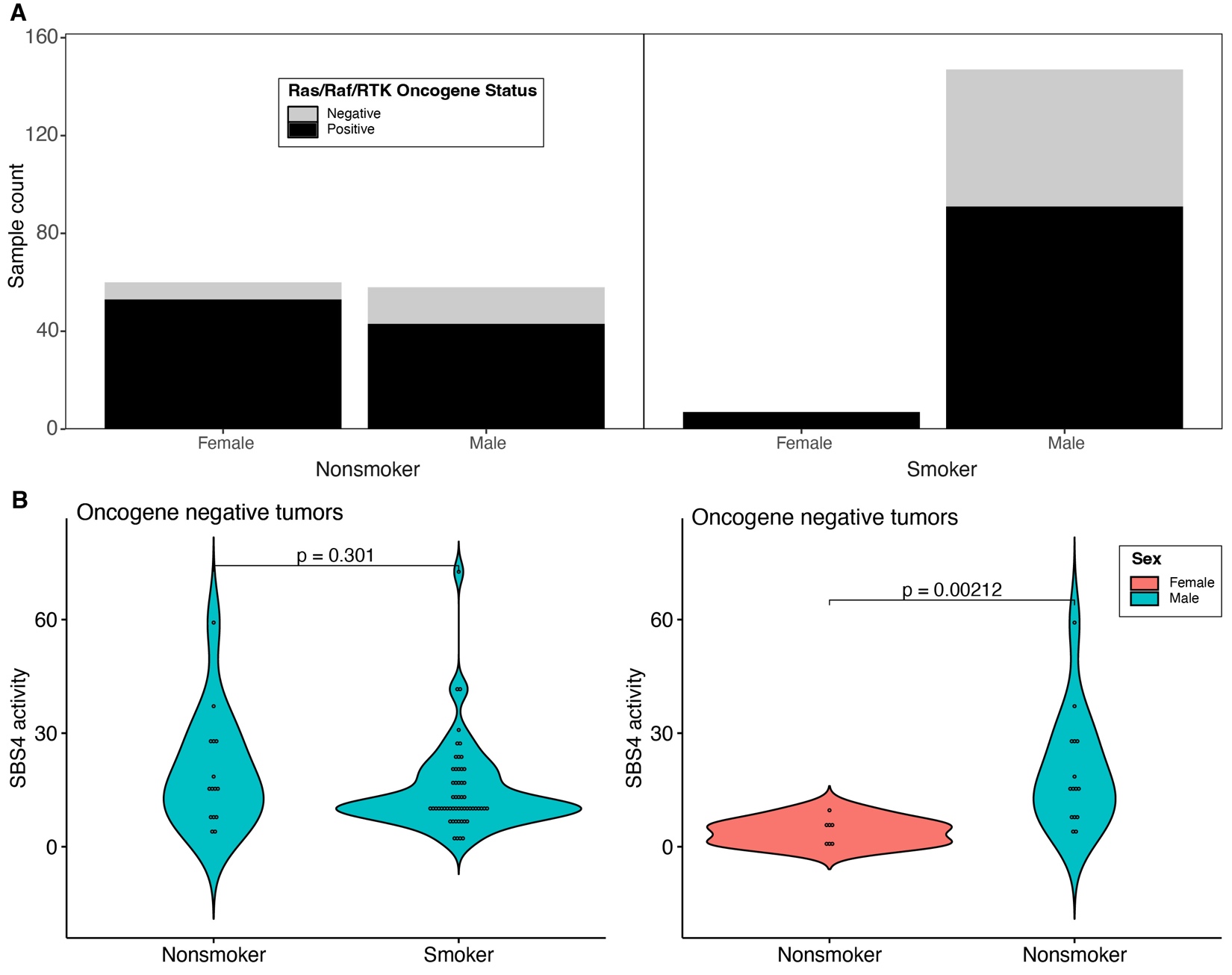
**

**Supplementary Figure 3. SBS4 activity associated with sex in oncogene negative tumors.** **(A)** The relationship between smoking status, sex, and oncogene status is shown for a subset of LUADs with complete clinical information (n=272). Tumors were considered “oncogene positive” if they had a mutation in the Ras/Raf/Receptor Tyrosine Kinase (RTK) pathway. A higher proportion of males were observed in smokers compared to non-smokers and a higher proportion of oncogene positive tumors were observed in females compared to males in both smokers and non-smokers. All female smokers were oncogene positive. **(B)** SBS4 activity was not significantly different between smoker males and nonsmokers males within oncogene negative LUADs using a Wilcoxon rank-sum test. SBS4 activity among oncogene negative LUADs was significantly higher in male non-smokers compared to female non-smokers in oncogene negative tumors. The same analysis cannot be done for smokers due to lack of female smokers without an oncogenic mutation.

**
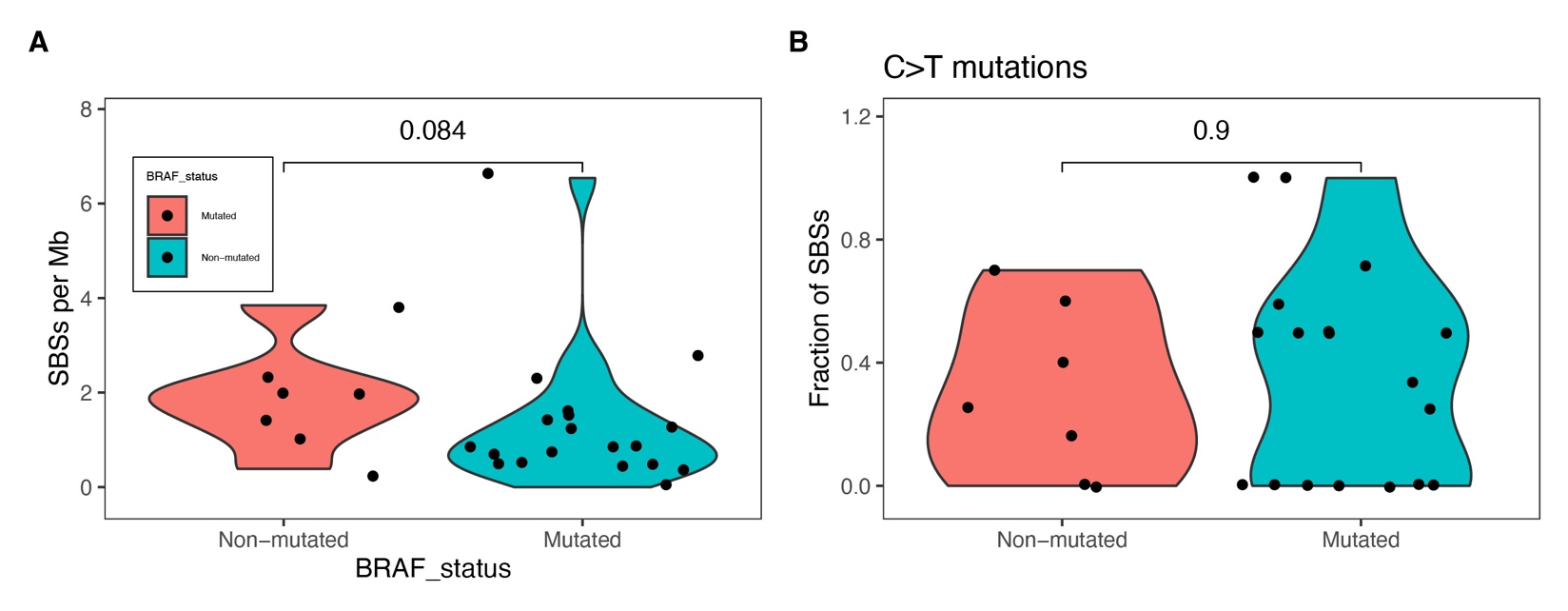
**

**Supplementary Figure 4. Association of SBS rates and C>T mutations with BRAF status in cutaneous melanomas.** No significant associations were found between *BRAF* mutation status and **(A)** total number of single base substitutions (SBS) per megabase or **(B)** the fraction of C>T mutations within each tumor.
